# Effect of Recombinant Gonadotropin on Testicular Function and Testicular Sperm Extraction in Five Cases of *NR0B1* (*DAX1*) Pathogenic Variants

**DOI:** 10.3389/fendo.2022.855082

**Published:** 2022-03-30

**Authors:** Jordan Teoli, Vincent Mezzarobba, Lucie Renault, Delphine Mallet, Hervé Lejeune, Pierre Chatelain, Frédérique Tixier, Marc Nicolino, Noël Peretti, Sandrine Giscard D’estaing, Béatrice Cuzin, Frédérique Dijoud, Florence Roucher-Boulez, Ingrid Plotton

**Affiliations:** ^1^ Service de Biochimie et Biologie Moléculaire, UM Pathologies Endocriniennes, CR DEV-GEN, Centre de Biologie et Pathologie Est, Hospices Civils de Lyon, Bron, France; ^2^ Université Claude Bernard Lyon 1, Lyon, France; ^3^ Institut Cellule Souche et Cerveau (SBRI), Unité INSERM, Centre de Recherche INSERM, Bron, France; ^4^ Fédération d’Endocrinologie, Hôpital Louis Pradel, Hospices Civils de Lyon, Bron, France; ^5^ Service de Médecine de la Reproduction, Hôpital Femme-Mère-Enfant, Hospices Civils de Lyon, Bron, France; ^6^ Service d’Endocrinologie Pédiatrique, Hôpital Femme Mère Enfant, Hospices Civils de Lyon, Bron, France; ^7^ Service de Gastroentérologie, Hépatologie et Nutrition Pédiatriques, Hôpital Femme Mère Enfant, Hospices Civils de Lyon, Bron, France; ^8^ Chirugie Urologique, Centre Lyonnais d’Urologie Bellecour, Lyon, France; ^9^ Service d’Anatomie Pathologique, Centre de Biologie et de Pathologie Est, Hospices Civils de Lyon, Bron, France

**Keywords:** testicular biopsy, adrenal hypoplasia, hypogonadotrophic hypogonadism, spermatogenesis, gonadotropin, congenital, male infertility, adrenal insufficiency

## Abstract

**Background:**

*NR0B1* pathogenic variants can cause congenital adrenal hypoplasia or primary adrenal insufficiency in early childhood usually associated with hypogonadotropic hypogonadism. *NR0B1* is necessary for organogenesis of the adrenal cortex and to maintain normal spermatogenesis. In humans, restoration of fertility in patients carrying *NR0B1* pathogenic variants is challenging.

**Objective:**

The aim of the study was to investigate the clinical, hormonal, histological, spermiological, and molecular genetic characteristics of a cohort of patients with *NR0B1* pathogenic variants, monitored for fertility preservation.

**Patients:**

We included five patients, including four teenagers, with *NR0B1* pathogenic or likely pathogenic variants. They all had primary adrenal insufficiency and were receiving replacement therapy with glucocorticoids and mineralocorticoids. Patients received recombinant follicle-stimulating hormone and recombinant human chorionic gonadotropin in order to induce spermatogenesis. Combined gonadotropin treatment was initiated between 13 years and 15 years and 6 months for the four teenagers and at 31 years and 2 months for the only adult. Physical and hormonal assessments were performed just before starting gonadotropin treatment. After 12 months of gonadotropin treatment, physical examination and hormonal assessments were repeated, and semen analyses were performed. If no sperm cells were observed in at least 2 semen collections at 3-month interval, testicular biopsy for testicular sperm extraction was proposed.

**Results:**

Bilateral testicular volume increased from 8 ml (interquartile range, 6–9) to 12 ml (10–16) after gonadotropin treatment. Inhibin B levels were relatively stable: 110 ng/L (46–139) before and 91 ng/L (20–120) at the end of gonadotropin treatment. Azoospermia was observed in all semen analyses for all cases during gonadotropin treatment. Three patients agreed to testicular biopsy; no mature sperm cells could be retrieved in any.

**Conclusion:**

We characterized a cohort of patients with *NR0B1* pathogenic or likely pathogenic variants for fertility preservation by recombinant gonadotropin treatment, which began either at puberty or in adulthood. No sperm cells could be retrieved in semen samples or testicular biopsy even after gonadotropin treatment, indicating that gonadotropin treatment, even when started at puberty, is ineffective for restoring fertility.

## 1 Introduction

X-linked adrenal hypoplasia congenita (X-AHC) is a pathology characterized by primary adrenal insufficiency (Addison’s disease), with onset most often at birth or in early childhood, and frequently associated with hypogonadotropic hypogonadism and spermatogenesis failure detected after puberty (1–6). Adrenal insufficiency can be treated effectively by glucocorticoid and mineralocorticoid replacement therapy. However, and contrary to other forms of hypogonadotropic hypogonadism (7), restoration of spermatogenesis remains challenging in X-AHC patients with azoospermia because of an added primary testicular injury in X-AHC (5, 6). Azoospermia in X-AHC patients classically does not respond to gonadotropin treatment, and no sperm cells were obtained in semen or even in testicular biopsies after this therapy (5, 6, 8–12) except in one study (13).

X-AHC is related to *NR0B1* (or *DAX1)* gene alterations. *NR0B1* is a gene with only two exons, carried by the X chromosome, belonging to the nuclear hormone receptor superfamily (1). It encodes a 470-amino acid (AA) protein with a suspected ligand-binding domain in the carboxyl-terminal portion and a 3.5-fold repeated motif responsible for protein–protein interactions in the amino-terminal portion (1, 4). This protein appears essential for organogenesis of the adrenal cortex, gonadal sex determination, development of the hypothalamic-pituitary-gonadotropic axis, and spermatogenesis (2, 3). Pathogenic variants in the *NR0B1* gene occur in 1:70,000 to 1:600,000 boys (14). More than one hundred pathogenic variants in the *NR0B1* gene have been described (4, 14, 15). Duplication of *NR0B1* is related to 46,XY sex reversal whereas deletion, indel or frameshift, splice sites, and nonsense or missense pathogenic variants are responsible for X-AHC in humans (2, 4, 14).

Here, we describe the impacts of pathogenic or likely pathogenic *NR0B1* variants on clinical, hormonal, histological, and spermiological aspects and on gonadotropin treatment response in five male patients, including four teenagers. Three of the five variants explored here have never been reported, whether in the literature or in databases.

## 2 Materials and Methods

### 2.1 Patients

This retrospective study included male patients monitored in the reproductive medicine department of Lyon University Hospital for fertility preservation. Patients were included if they had received a gonadotropin treatment after 2010 and if a hemizygous *NR0B1* pathogenic or likely pathogenic variant was identified in the molecular endocrinology unit of the laboratory using Sanger sequencing on DNA extracted from whole blood. Variants were described using reference NP_000466.2 for DAX1 protein and NM_000475.5 for *NR0B1* transcript on GRCh37/hg19 human genome assembly. Pathogenic or likely pathogenic classification was based on the American College of Medical Genetics and Association of Medical Pathologists (ACMG) consensus recommendation (16) with the help of the Gnomad_v2 (https://gnomad.broadinstitute.org/), ClinVar (https://www.ncbi.nlm.nih.gov/clinvar/), and dbSNP databases (https://www.ncbi.nlm.nih.gov/snp/) and *in silico* prediction tools listed by Mobidetails (an online DNA variant interpretation tool) (17).

Patients and, as appropriate, their parents signed an informed written consent form for genetic study. In accordance with French legislation, review board submission was not required, owing to the observational nature of the study. The study was conducted in accordance with the principles of the Declaration of Helsinki.

### 2.2 Protocol

During the fertility preservation procedure, history, psychological evaluation, and clinical data were recorded systematically, including physical examination at each visit: before treatment and the day of the semen analysis and/or blood sampling for hormonal analysis.

At the time of inclusion, bilateral testicular volume (BTV: sum of right and left testis volume, considered normal if ≥30 ml) was assessed using a Prader orchidometer. Blood samples were taken for follicle-stimulating hormone (FSH), luteinizing hormone (LH), total testosterone, anti-Müllerian hormone (AMH), and inhibin B assay (pretherapeutic assessment).

Patients included in the fertility preservation protocol received a combination of recombinant FSH (rFSH) and recombinant human chorionic gonadotropin (rhCG). The gonadotropin treatment was increased gradually to reach a subcutaneous injection dose of 150 IU rFSH three times per week and 1,500 IU rhCG twice a week. During follow-up visits, rFSH dosage was adapted to inhibin B level and rhCG dosage to testosterone level.

**Figure 1:**
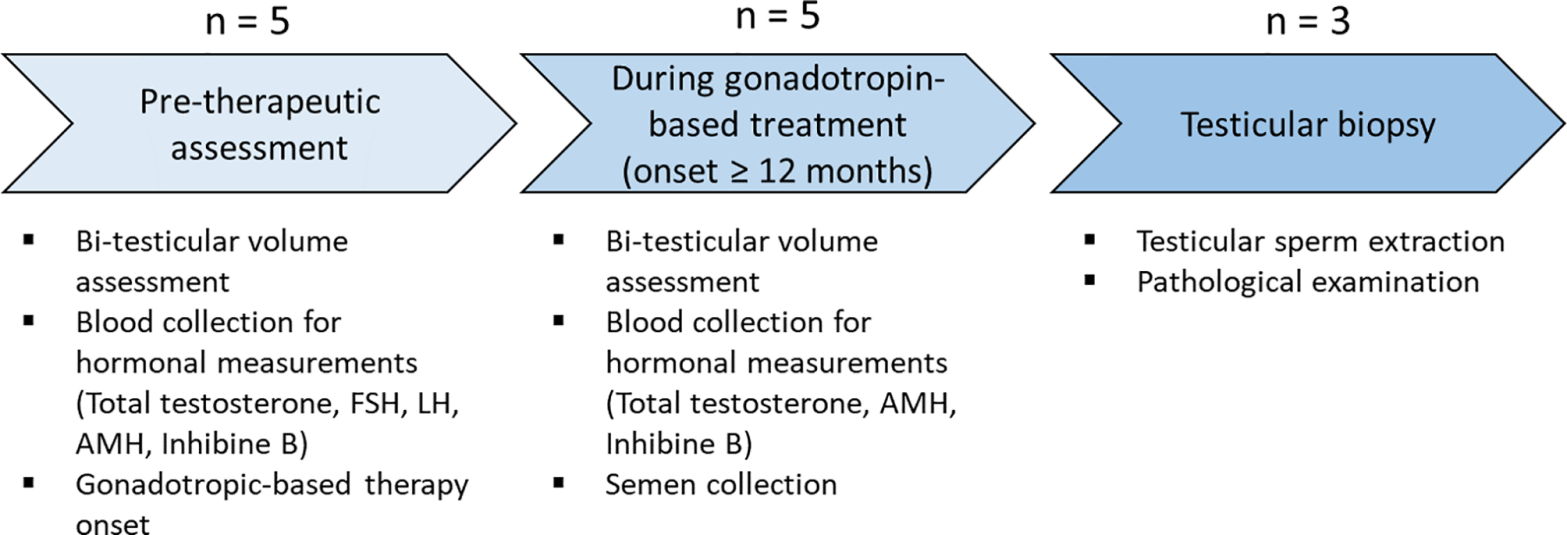
Protocol design. Physical examination and biological samples at each step of the protocol. The number of patients undergoing each step is indicated. Each step of the protocol is represented by an arrow. AMH, anti-Müllerian hormone; FSH, follicle-stimulating hormone; LH, luteinizing hormone.

After 12- to 42-month gonadotropin treatment, BTV was reassessed and blood samples were repeated for hormonal measurement (total testosterone, AMH, inhibin B). Semen was collected under gonadotropin treatment for laboratory analysis. Testicular biopsy for testicular sperm extraction (TESE) was proposed if azoospermia was observed on two semen analyses 3 months apart (**Figure 1**).

### 2.3 Laboratory Assays

#### 2.3.1 Hormonal Measurements

Plasma FSH and LH were assessed by an automated chemiluminescence immunometric assay on Architect i2000SR (Abbott, Chicago, IL, USA). The interassay coefficient of variation (CVb) was ≤3.9% at 6, 20, and 40 IU/L and ≤4.2% at 4, 14, and 44 IU/L for FSH and LH, respectively. The limit of quantification (LOQ) was 0.05 IU/L for FSH and LH. In men with normal testicular function, normal ranges extended from 1.1 to 7.2 IU/L and 1.3 to 5.8 IU/L for FSH and LH, respectively.

Plasma total testosterone was assessed on in-house liquid chromatography coupled with tandem mass spectrometry after supported liquid–liquid extraction using diatomaceous earth. CVb was ≤7.8% at 1.96, 7.50, 8.20, and 23.46 nmol/L. LOQ was 0.13 nmol/L. Normal ranges were 10.40 to 26.00 nmol/L in young men and 0.28 ± 0.01 nmol/L (mean ± standard deviation) in prepubescent boys.

Serum AMH was assessed by automated electrochemiluminescence assay on Cobas e601 (Roche Diagnostics, Basel, Switzerland). CVb was ≤2.8% at 5.5 and 30 pmol/L. LOQ was 0.21 pmol/L. Normal ranges extended from 15 to 60 pmol/L in men with normal spermatogenesis (18).

Serum inhibin B was assessed by enzyme immunoassay using the Inhibin B Gen II ELISA kit (Beckman Coulter, Brea, CA, USA). CVb was ≤13.7% at 115 and 420 ng/L. LOQ was 5 ng/L. Normal lab ranges for men with normal testicular function were taken from the normozoospermic cohort of Pierik et al.: 55 to 309 ng/L (19) and, for boys aged 12–17 years were taken from Crofton et al.: 74 to 470 ng/L (20).

#### 2.3.2 Semen Analysis

Semen collection was carried out by masturbation in the reproduction laboratory after 3–5 days of sexual abstinence. Analysis was performed according to the 2010 World Health Organization criteria (21).

#### 2.3.3 Testicular Biopsy and Conventional TESE

The multiple bilateral testicular biopsies and conventional TESE procedure was as previously described (22). TESE was performed in the reproduction unit of the laboratory.

#### 2.3.4 Histological Analysis

In parallel, some biopsy fragments were sent to the pathology unit of the laboratory, fixed in alcohol, formalin, and acetic acid (AFA) and paraffin embedded. Three-micrometer slices were stained by hematoxylin–phloxin–saffron.

Slide evaluation was performed on a Leica DM2500 microscope. All tubules within five image fields were evaluated. The presence of a lumen and the most advanced germ cell were noted. Germ cells were identified on the basis on their morphology (size and shape) and location (23). The number of Leydig cells was estimated on three fields at ×40 magnification.

### 2.4 Statistical Analysis

Quantitative data were expressed as median (interquartile range). Values below LOQ were considered equal to the LOQ. Analysis used R software v3.6.3 (R Foundation for Statistical Computing, Vienna, Austria).

## 3 Results

Five patients from unrelated families were included in the study. A primary adrenal insufficiency crisis occurred at birth in three patients (patients 1, 2, and 4) and in childhood up to 10 years of age in the other two (patients 3 and 5). Patients started glucocorticoid and mineralocorticoid replacement therapy. Genetic analysis found *NR0B1* variants that confirmed X-AHC diagnosis.

**Figure 2:**
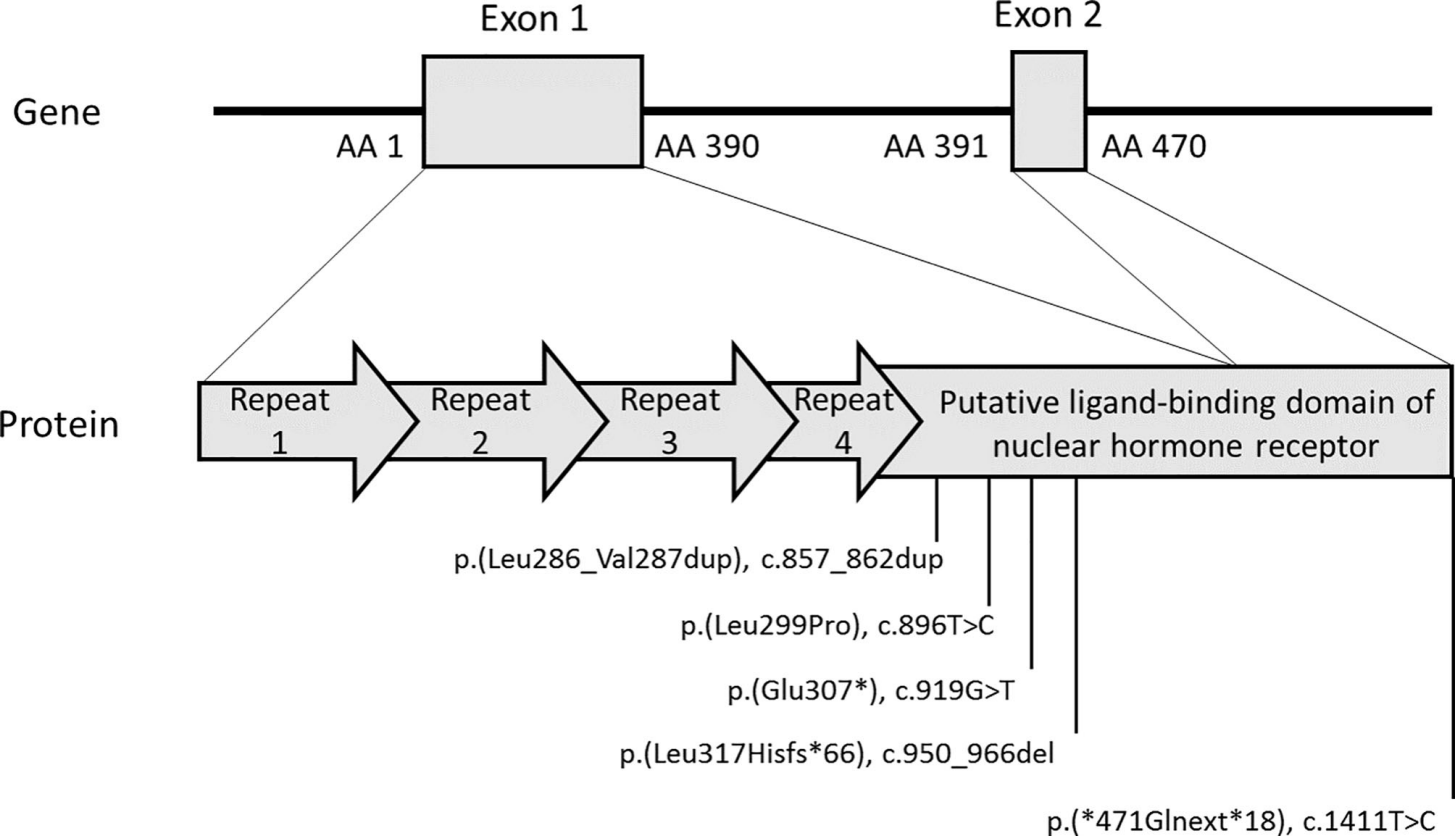
*NR0B1* variants identified in patients. The figure depicts the two exons of the *NR0B1* gene confronted with the 470 AA DAX1 protein. The DAX1 protein diagram resumes the 3.5-fold repeated motif in the amino-terminal portion (arrows) and the putative ligand-binding domain of nuclear hormone receptor in the carboxyl-terminal portion. The five variants identified in the five patients are positioned on the protein diagram. All the reported variants cluster in the putative ligand-binding domain of nuclear hormone receptor. Variants were described using reference NP_000466.2 for DAX1 protein and NM_000475.5 for *NR0B1* transcript on GRCh37/hg19 human genome assembly. AA, amino acid.

Locations of patients’ *NR0B1* variants are reported in **Table 1** and displayed in **Figure 2**. Based on the ACMG criteria, all the variants were considered likely pathogenic or pathogenic (**Table 1**). Two of the five variants were previously reported elsewhere. The variant NM_000475.5: c.1411T>C p.(*471Glnext*18) (patient 3) was recently reported in the literature and considered likely pathogenic in ClinVar. The variant NM_000475.5: c.919G>T p.(Glu307*) (patient 4) was reported in dbSNP and considered pathogenic in ClinVar. The other three variants had not been reported in the literature, ClinVar, or dbSNP. None of the variants were reported in Gnomad_v2. All variants were clustered in the putative ligand-binding protein domain (**Figure 2**).

**Table 1:** Patients’ characteristics.

	Patient	1	2	3	4	5
Diagnosis	Age at diagnosis	At birth	At birth	10 years	At birth	6 years
	Diagnostic context	Acute primary adrenal insufficiency	Acute primary adrenal insufficiency	Acute primary adrenal insufficiency	Acute primary adrenal insufficiency	Acute primary adrenal insufficiency
Pretherapeutic assessment (at age of gonadotropin initiation)	Bilateral testicular volume (ml)	10	9	6	8	6
	FSH/LH (IU/L)	1.8/0.4	4.8/0.8	2.2/0.91	0.7/0.07	NA
	Total testosterone (nmol/L)	3.44	1	0.16	<0.13	NA
	AMH (pmol/L)	414.1	58.6	249.5	192.9	46.4
	Inhibin B (ng/L)	139	46	187	110	42
Gonadotropin treatment	Age of gonadotropin treatment initiation	13y11m	15y6m	13y	14y	31y2m
	Age of gonadotropin treatment termination	16y7m	18y8m	15y11m	17y	34y8m
	Gonadotropin treatment	rFSH + rhCG	rFSH + rhCG	Priming rFSH for 6 months then rFSH + rhCG	rFSH + rhCG	rFSH + rhCG after 15 years of testosterone supplementation
	Total duration of gonadotropin treatment	32 months	38 months	35 months	36 months	42 months
Assessment during gonadotropin treatment or the earliest assessment after termination	Age at assessment	16y7m	20y11m	15y8m	15y	34y8m
	Bilateral testicular volume (ml)	10	12	20	16	7
	Total testosterone (nmol/L)	16.82	NA	12.60	15.51	NA
	AMH (pmol/L)	60.4	15.2	41.0	105.4	36.9
	Inhibin B (ng/L)	91	<5	214	120	20
Semen collection	Result	Azoospermia On 4 samples	Azoospermia On 2 samples	Azoospermia On 1 sample	Azoospermia On 3 samples	Azoospermia On 3 samples
	Time of sampling since start of therapy	Between 12 and 32 months	At 35 and 38 months	At 35 months	Between 12 and 31 months	Between 16 and 42 months
TESE	Result	No sperm cells retrieved	Not done	Not done	No sperm cells retrieved	No sperm cells retrieved
	Time of testicular biopsy since start of therapy	32 months	NA	NA	31 months	42 months
	Age at biopsy	16y7m	NA	NA	16y7m	34y8m
*NR0B1* variant (NP_000466.2, NM_000475.5, GRCh37/hg19)	Location	p.(Leu286_Val287dup) c.857_862dup	p.(Leu299Pro) c.896T>C	p.(*471Glnext*18) c.1411T>C	p.(Glu307*) c.919G>T	p.(Leu317Hisfs*66) c.950_966del
	ACMG class (ACMG criteria)	Likely pathogenic (PM1, PM2, PM4, PP3, PP4)	Likely pathogenic (PM1+PM2+PP3 PP4)	Likely pathogenic (PM2, PM4, PP3, PP4, PP5)	Pathogenic (PVS1, PM1, PM2, PP3, PP4, PP5)	Pathogenic (PVS1, PM1, PM2, PP4)

AA, amino acid; ACMG, American College of Medical Genetics and Genomics; AMH, anti-Müllerian hormone; FSH, follicle-stimulating hormone; LH, luteinizing hormone; NA, not applicable; rFSH, recombinant follicle-stimulating hormone; rhCG, recombinant human chorionic gonadotropin; TESE, testicular sperm extraction.

ACMG criteria: pathogenic moderate (PM); pathogenic supporting (PP); pathogenic strong (PS); pathogenic very strong (PVS).

“PVS1: null variant (nonsense, frameshift, canonical +/−1 or 2 splice sites, initiation codon, single or multiexon deletion) in a gene where loss of function is a known mechanism of disease.”

“PM1: located in a mutational hotspot and/or critical and well-established functional domain (e.g., active site of an enzyme) without benign variation.”

“PM2: absent from controls (or at extremely low frequency if recessive) in Exome Sequencing Project, 1,000 Genomes or ExAC.”

“PM4: protein length changes due to in-frame deletions/insertions in a nonrepeat region or stop-loss variants.”

“PP3: multiple lines of computational evidence support a deleterious effect on the gene or gene product (conservation, evolutionary, splicing impact, etc.).”

“PP4: patient’s phenotype or family history is highly specific for a disease with a single genetic etiology.”

“PP5: reputable source recently reports variant as pathogenic but the evidence is not available to the laboratory to perform an independent evaluation.”

*Patient 1* carried the novel variant NM_000475.5: c.857_862dup p.(Leu286_Val287dup), which adds two additional AA in an alpha helix of the putative ligand-binding domain of nuclear hormone receptor of *NR0B1*. His mother was heterozygous for the variant. His healthy brother did not carry the variant. He had one infertile maternal uncle and one maternal uncle who died at the age of 1 month of life.

*Patient 2* was hemizygous for the novel substitution NM_000475.5: c.896T>C p.(Leu299Pro), inherited from his heterozygous mother. His healthy brother did not carry the variant.

*Patient 3* had a stop-loss variant NM_000475.5: c.1411T>C p.(*471Glnext*18), which extended the C-terminal portion of the protein by 18 additional AA. Genetic analysis was not performed on his mother, but he had two healthy brothers who did not carry the variant. He had one maternal uncle with adrenal insufficiency.

*Patient 4* had a stop gain variant NM_000475.5: c.919G>T p.(Glu307*), leading to loss of part of the putative ligand-binding domain of the nuclear hormone receptor of *NR0B1*. His mother was heterozygous for the variant. His healthy brother did not carry the variant.

*Patient 5* carried a new frameshift variant NM_000475.5: c.950_966del p.(Leu317Hisfs*66) caused by a 17-bp deletion. Genetic analysis was not performed on his mother, and family history was not available.

These patients were enrolled in a fertility preservation protocol based on gonadotropin treatment after 2010: four at puberty (patients 1 to 4) and one in adulthood (patient 5). FSH, LH, and plasma total testosterone levels assessed in four of the five patients (patients 1 to 4) immediately before starting gonadotropin treatment showed low testosterone levels (0.58nmol/L [0.15–1.61]) compared with FSH (2.00 IU/L [1.53–2.85]) and LH concentrations (0.60 IU/L [0.32–0.83]), which were not elevated. BTV was low (8 ml [6–9]). AMH levels were high in three patients. Inhibin B levels were low or near the lower limit of normal (110 ng/L [46–139]) (**Table 1**).

Patients then received combined gonadotropin treatment. Patient 5 had received testosterone therapy for 15 years before starting the gonadotropin treatment, and patient 3 received only rFSH during the first 6 months (priming rFSH) of gonadotropin treatment.

**Figure 3:**
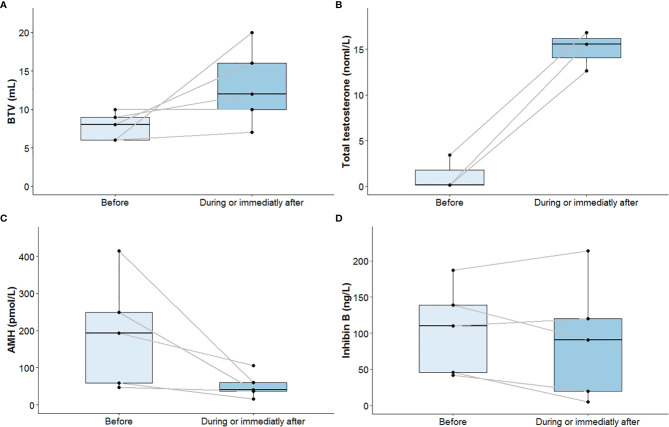
Bi-testicular volume **(A)**, plasma total testosterone **(B)**, serum AMH **(C)**, and serum inhibin B **(D)** progression before and during or immediately after termination of gonadotropin treatment. Median values and interquartile ranges are presented in two boxplots for each parameter: one for values measured before initiation of gonadotropin treatment and one for values measured during or immediately after termination. Each black dot corresponds to one patient. Black dots obtained from the same patient before and during or immediately after termination of gonadotropin treatment are connected by solid gray lines. For plasma total testosterone, only patients with values before and during or immediately after termination of gonadotropin treatment were considered. An increase in BTV and total testosterone levels and a decrease in AMH levels after at least 12 months under gonadotropin treatment is observed. Variation in inhibin B levels is not as clear. AMH, anti-Müllerian hormone; BTV, bi-testicular volume.

After at least 12 months under gonadotropin treatment, physical and hormonal assessments were repeated. BTV rose from 8 ml (6–9) before to 12 ml (10–16) after gonadotropin treatment, and values remained very low. Total testosterone increased from 0.58 nmol/L (0.15–1.61) to normal adult values at 15.51 nmol/L (14.07–16.17). AMH levels decreased from 192.9 pmol/L (58.6–249.5) before to 41 pmol/L (36.9–60.4) after gonadotropin treatment. AMH levels approached usual values after gonadotropin treatment. Inhibin B levels stayed quite stable: 110 ng/L (46–139) at the beginning to 91 ng/L (20–120) at the end of gonadotropin treatment and remained quite low (**Table 1** and **Figure 3**).

Semen analysis was performed after at least 12 months of gonadotropin treatment. No sperm cells were retrieved in any of the five patients. Four patients (patients 1, 2, 4, and 5) were eligible for TESE, since azoospermia was observed on two semen analyses 3 months apart, whereas only one semen analysis was performed in the other patient (patient 3). Only three patients (patients 1, 4, and 5) agreed to testicular biopsy, and the TESE procedure was negative for all: no spermatozoa could be extracted and cryopreserved (**Table 1**).

Histological analysis of testicular biopsies showed pubescent testicular parenchyma with severe hypospermatogenesis lesions and maturation arrest. In all three patients, interstitial tissue was edematous and there was no dysplasia.

**Figure 4:**
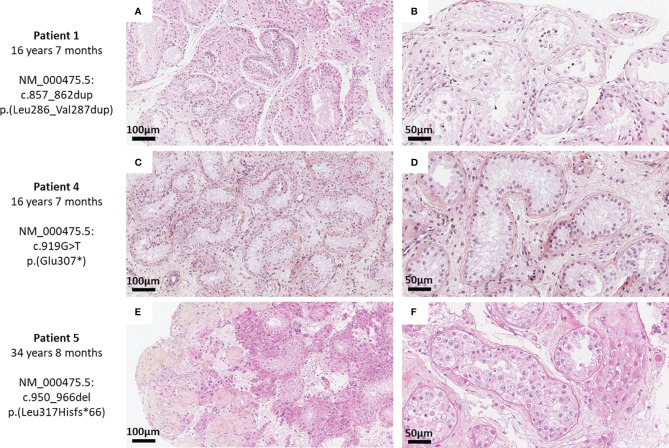
Histological sections of testicular biopsies of patient 1 **(A**, **B)**, patient 4 **(C**, **D)**, and patient 5 **(E, F)** after gonadotropin treatment. Histological sections of testicular biopsies are shown at magnifications ×200 **(A, C, E)** and ×400 **(B, D, F)** after a hematoxylin–phloxin–saffron staining. All biopsies showed severe hypospermatogenesis with either Sertoli cell-only profile **(C**, **D)**, histological mosaicism profile with Leydig cell hyperplasia **(A**, **E)**, or maturation arrest at round spermatid stage **(B)** or spermatocyte stage **(F)**.

Histological examination in patient 1 (16y7m) showed severe hypospermatogenesis with maturation arrest (histological mosaicism profile) associated with Leydig cell hyperplasia. Most of the seminiferous tubules showed Sertoli cell-only syndrome. Some tubules showed incomplete spermatogenesis with a few spermatocytes and round spermatids but no mature germ cells. Rare prepubertal tubules were observed, without a central lumen and with very rare spermatogonia (**Figures 4A, B**).

Patient 4 (16y7m) showed pubescent testis with Sertoli cells only. The stroma was edematous, with some Leydig cells but without hyperplasia (**Figures 4C, D**).

Patient 5 (34y8m) showed severe hypospermatogenesis with numerous tubules with Sertoli cells only and some tubules with maturation arrest at spermatocyte level. Nodular Leydig cell hyperplasia was also observed. The tubules were surrounded by thickened lamina propria and some were totally atrophic (**Figures 4E, F**).

## 4 Discussion

We characterized a cohort of patients with pathogenic or likely pathogenic *NR0B1* variants, for fertility preservation associated to gonadotropin treatment, at onset of puberty in four cases and in adulthood for the other. To our knowledge, this is the first time that the impact of gonadotropin treatment has been reported in literature in adolescents.

Here, X-AHC was diagnosed during acute primary adrenal insufficiency at birth or in childhood up to 10 years of age. Variants were classified as pathogenic or likely pathogenic; all were located in a hotspot: the putative ligand-binding domain of nuclear hormone receptor. All variants modified the length of the protein if one was produced, except the missense variant NM_000475.5: c.896T>C p.(Leu299Pro). Leu299 is included in the same highly LLxLxLx-conserved domain as Leu295 and Leu297, substitution of which in Proline was reported in X-AHC patients (9, 24). This highlighted the potential deleterious effect of p.(Leu299Pro) for DAX1 protein function. The variant NM_000475.5: c.1411T>C p.(*471Glnext*18) was reported in an X-AHC boy with precocious puberty (25). Protein–protein docking showed the addition of 18 additional AA after the stop codon decreased interaction between DAX1 protein and SF1 protein. The boy was followed up from 11 to 15.1 years of age; we do not know whether he would develop hypogonadotropic hypogonadism later. No semen collection was performed, but testicular volume was 3 ml bilaterally and inhibin B levels were low, suggesting a spermatogenesis defect (25). In contrast, the patient carrying NM_000475.5: c.1411T>C p.(*471Glnext*18) (patient 3) did not present precocious puberty and had subnormal BTV of 20 ml and normal inhibin B level at 15 years and 8 months.

Patients with frameshift variant or stop-gain variant might have been expected to have earlier revelation of the pathology than those with missense variant or in-frame insertion. However, hormonal levels, BTV, age at diagnosis, and gonadotropin treatment response showed no correlations with the molecular variant. This confirms the absence of any clear genotype–phenotype relation in X-AHC patients and the heterogeneity of the pathology reported elsewhere (4, 10, 12, 26, 27).

Low levels of total testosterone in parallel to the defect of increased FSH and LH levels indicated that patients presented hypogonadotropic hypogonadism. Inhibin B is secreted by Sertoli cells, and BTV and inhibin B are markers of spermatogenic potential (28–30). Therefore, reduced BTV and low or low-normal inhibin B values for age suggest spermatogenesis failure which can be integrated in the hypogonadotropic hypogonadism profile and in the primary gonadal defect reported in X-AHC patients (5, 9, 10, 13). As observed here, some authors reported testicular volume ranging from 3 to 6 ml bilaterally in most X-AHC cases (6, 9, 11, 25, 26, 31, 32). However, testicular volume may be normal in mild forms of X-AHC (10). Likewise, inhibin B was reported to be low or in the lowest range of normal at puberty or after (6, 10, 25, 26, 31, 33).

After gonadotropin treatment, total testosterone levels increased and AMH levels decreased. This indicates that Leydig cells retain their cellular function and ability to be stimulated by gonadotropins in X-AHC patients. The level of testosterone secretion stimulation by hCG in X-AHC patients varies from case to case (5, 9, 11–13, 34). It is already known physiologically that testosterone induces maturation of Sertoli cells, which express the androgen receptor, manifested by a stop in their multiplication and a sharp decrease of their AMH secretion (35, 36). Consequently, the decrease in AMH observed in our patients could suggest the presence of mature Sertoli cells which express the androgen receptor in X-AHC patients.

After gonadotropin treatment, we also showed an overall increase in BTV, although it remained below the normal range, suggesting a modest increase in germ cells. BTV did not respond to treatment in the adolescent patient where it was highest before the start of the gonadotropin treatment (patient 1) or in the patient treated in adulthood (patient 5). Interestingly, the greatest increase in BTV was in the patient who received rFSH alone (priming rFSH) during the first 6 months (patient 3). As reported by Dwyer et al. in congenital hypogonadotropic hypogonadism due to GnRH defect, priming rFSH treatment can increase the Sertoli cell population before testosterone secretion (induced by the addition of hCG) stops their multiplication (37). Unfortunately, patient 3 was not eligible for testicular biopsy and TESE to see if priming rFSH could improve his spermatogenesis. Inhibin B level variations were less clear, with a slight increase after gonadotropin treatment in some patients and a decrease in others, indicating a primary defect in Sertoli cell function. Our observations were consistent with other cases reported in literature which showed an increase in testicular volume (5, 13, 34) but no or only slight increase in inhibin B levels after gonadotropin therapy (9). However, there were some discrepancies. A study in an azoospermic 36-year-old man showed a rise in inhibin B after 5 months of combined gonadotropin treatment, but in a mild form of X-AHC (10), and another study reported no significant increase in testicular volume after gonadotropin treatment in seven adult patients (4.0 ± 2.9 vs. 4.9 ± 3.3 ml) (8). In any case, inhibin B response to gonadotropin treatment, as BTV response, was generally much lower than in other forms of hypogonadotropic hypogonadism such as Kallmann syndrome (7, 38), which reinforces the idea of a peripheral gonadal defect in X-AHC.

Some authors reported progressive degradation of the hypothalamic-pituitary-gonadal axis with age in X-AHC patients. Galeotti et al. reported a cohort of eight X-AHC patients with normal minipuberty (27). Others described infants with normal or increased testicular volume for age and a physiologic minipuberty whereas the maternal uncle, bearing the same *NR0B1* variant, failed to enter puberty (32, 39). In terms of fertility, a mutated *NR0B1* murine model suggested that spermatogenesis may deteriorate gradually with age (40). This was supported in humans in a mild form of X-AHC in which inhibin B decreased from 148 ng/L at 35 years to 38 ng/L at 43 years (normal range, 80–270 ng/L), sperm count decreased from 4 million at 23 years to 0.05 million at 37 years, and testicular volume decreased from 20 ml bilaterally at 32 years to 15 ml at 47 years (10). Data from Galeotti et al. were also consistent with progressive degradation of spermatogenesis, with normal inhibin B values in the first year of life, decreasing in adolescence and adulthood according to the age-related normal ranges (27). Previous studies, using several combined gonadotropin drugs, doses, and treatment duration ranging from 5 months to 3 years, failed to restore spermatogenesis in X-AHC patients (5, 6, 9–12). None of these studies used priming rFSH. What these studies had in common was also that gonadotropin treatment was initiated in adulthood (5, 6, 8–12); it may therefore be advantageous to start gonadotropin treatment earlier in life. As inhibin B levels were not below the limit of quantification for any of our patients and in some cases BTV responded to gonadotropin therapy, we might expect to retrieve sperm cells in semen or testicular biopsy after TESE, but in fact failed to do so, whether gonadotropin treatment was started either in adulthood or at age of puberty. The gonadotropin treatment protocol we used may be contested, but a similar one allowed sperm cells to be retrieved from semen in almost the entire cohort of adults with hypogonadotropic hypogonadism (including 11 patients with Kallmann syndrome) after around 12 months of treatment on average (7). Inhibin B and BTV appear to be poorer biomarkers of spermatogenesis in X-AHC patients than in other forms of nonobstructive azoospermia, where elevation after gonadotropin treatment correlated with the presence of sperm cells in semen collection (7, 38).

Interestingly, some studies held out hope for X-AHC patients to be able to father children. Some cases of spontaneous paternity were reported, free of any drugs, but in mild forms of X-AHC. Vargas et al. reported a kindred with late-onset X-AHC where a man and his uncle had children at respectively 32 and 39 years of age, before diagnosis of primary adrenal insufficiency (26). The uncle was then totally azoospermic at 64 years of age. Raffin-Sanson et al. reported a man who had two sons, one at 35 years of age by *in vitro* fertilization and one naturally at 37 years of age. He was diagnosed with adrenal insufficiency at 19 years of age, and his sperm count decreased drastically with age (10). Otherwise, using a gonadotropin supplementation protocol almost identical to ours (administration of menotropin consisting of 150 IU FSH and 150 IU LH three times per week, combined with administration of 1,500 IU hCG two times per week for 20 months), Frapsauce et al. succeeded in retrieving sperm cells from a 25-year-old man with a classic form of X-AHC (adrenal crisis at 3 weeks of life) by testicular sperm extraction, with intracytoplasmic sperm injection resulting in the birth of a heathy child (13).

In the present study, testicular biopsies showed severe hypospermatogenesis with the absence of mature germ cells, although biopsy was realized during adolescence for two patients and a combined gonadotropin treatment was used. Spermatogonia and hyperplastic Leydig cells were seen in two of the three patients with testicular biopsy (one with biopsy during adolescence and the other during adulthood). Sertoli cell injury was found in all patients. These observations are consistent with the absence of sperm cells in semen or TESE after gonadotropin therapy, and with decreased inhibin B levels and reduced testicular volume unresponsive or poorly responsive to gonadotropin treatment. Histological examination in a murine model with mutated *NR0B1* highlighted a progressive degeneration of seminiferous tubules with hyperplastic Leydig cells and failure to maintain germ cells (40). In humans, Seminara et al. showed a Sertoli cell-only syndrome with scarce spermatogonia not maturing into sperm cells in a 27-year-old man treated with hCG for 7 years (5). In a 20-year-old man with X-AHC, testicular biopsy found a disorganized structure of seminiferous tubules with moderate hyperplastic Leydig cells and proliferative interstitial tissue after 6 months of gonadotrophin treatment (6), in line with the abnormalities found in the present patients. Interestingly, postmortem histological testicular examination of a newborn baby who had a mutated *NR0B1* and died of adrenal crisis at 23 days showed physiologic testicular histology for age with numerous Sertoli cells and numerous spermatogonia (9). Normal testicular histology was also described in a 9-year-old boy. The structure of his seminiferous tubes was conserved, and they contained spermatogonia, while Leydig cells were not hyperplastic (41). In the 25-year-old patient with a classic form of X-AHC and who fathered a child, reported by Frapsauce et al., biopsy showed mostly incomplete spermatogenesis up to spermatocyte stage but very rare focal spermatogenesis leading to mature sperm cells for TESE-intracytoplasmic sperm injection (13). The discrepancy between the present TESE results and those of Frapsauce et al. may be due to the fact that their patient did not carry the same *NR0B1* variant as ours, and it is important to bear in mind the heterogeneous spectrum of X-AHC even in patients carrying the same *NR0B1* variant (4, 10, 26, 27). However, it cannot be excluded that focal spermatogenesis existed elsewhere in our patients’ testes but simply not in the multiple bilateral biopsied fragments.

## 5 Perspectives

Testicular biopsy failed to retrieve mature sperm cells although it was performed during adolescence in two patients under gonadotropin treatment, suggesting that testicular biopsy may be performed as early as possible (at the age of usual spermatogenesis onset) after diagnosis of X-AHC. If spermatogonia and functional seminiferous tubules can be retrieved from the testicular biopsy, X-AHC may be an indication for the emerging *in vitro* spermatogenesis technology (42), on the hypothesis that spermatogenesis defect in X-AHC patients is due to impaired Sertoli cell function. In 2016, Perrard et al. managed for the first time to perform complete spermatogenesis from culture of adult human seminiferous tubule segments, using a bioreactor in a specific culture medium (42). Earlier gonadotropin treatment, at onset of puberty (around 11 years of age) or even before the rise in intratesticular testosterone secretion, with priming rFSH to optimize Sertoli cell function, should also be investigated.

## 6 Conclusion

The present data extend our understanding of X-AHC, reporting three new *NR0B1* variants. These variants were associated with classic forms of X-AHC with azoospermia not responding to combined gonadotropin treatment. No sperm cells could be retrieved from semen collection or testicular biopsy even when gonadotropin treatment was started at the age of puberty. However, spermatogonia were seen in testicular biopsies of two out of three patients, holding out hope for X-AHC patients to father children using *in vitro* spermatogenesis technique currently in development.

## Data Availability Statement

The original contributions presented in the study are included in the article/supplementary material. Further inquiries can be directed to the corresponding authors.

## Ethics Statement

Ethical review and approval was not required for the study on human participants in accordance with the local legislation and institutional requirements. Written informed consent to participate in this study was provided by the participants’ legal guardian/next of kin.

## Author Contributions

VM, LR and JT wrote the manuscript. IP, DM, FD, and FR supervised the laboratory procedures. JT and VM performed the statistical analysis. JT, VM, FD, FR, and IP interpreted the data. Patient care was performed by HL, PC, FT, MN, NP, SGD, BC, and IP. All authors read, revised, and approved the final version of the manuscript.

## Conflict of Interest

The authors declare that the research was conducted in the absence of any commercial or financial relationships that could be construed as a potential conflict of interest.

## Publisher’s Note

All claims expressed in this article are solely those of the authors and do not necessarily represent those of their affiliated organizations, or those of the publisher, the editors and the reviewers. Any product that may be evaluated in this article, or claim that may be made by its manufacturer, is not guaranteed or endorsed by the publisher.

## Abbreviations

AA, amino acid; ACMG, American College of Medical Genetics and Genomics; X-AHC, X-linked adrenal hypoplasia congenita; AMH, anti-Müllerian hormone; BTV, bilateral testicular volume; CVb, interassay coefficient of variation; FSH, follicle-stimulating hormone; LBD, ligand-binding domain; LH, luteinizing hormone; LOQ, limit of quantification; rFSH, recombinant follicle-stimulating hormone; rhCG, recombinant human chorionic gonadotropin; TESE, testicular sperm extraction.
